# Outdoor activity time and depression risk among adults aged 40 years and older: a cross-sectional analysis of NHANES 2011–2018 data

**DOI:** 10.3389/fpsyg.2025.1506168

**Published:** 2025-01-22

**Authors:** Kai Liu, Cheng Guo, Juan Xie, Liming Cheng

**Affiliations:** ^1^Comprehensive Pediatrics, Kunming Children's Hospital, Kunming, China; ^2^Department of Anesthesiology, Kunming Children's Hospital, Kunming, China

**Keywords:** outdoor, depression, activities, time, NHANES

## Abstract

**Background:**

Depression is a significant global public health issue, affecting millions worldwide. Outdoor activities have shown potential mental health benefits, but the specific mechanisms and influencing factors remain unclear.

**Objectives:**

This study aimed to investigate the association between time spent outdoors and depression risk among U.S. adults, with a focus on variations across age and ethnic groups.

**Methods:**

Using data from the NHANES 2011–2018 survey, we analyzed 9,036 adults aged 20 years and older. Participants self-reported their outdoor activity time and depressive symptoms. Statistical analysis, accounting for various demographic and lifestyle factors, was employed to assess the relationship between outdoor activity and depression risk.

**Results:**

Spending more time outdoors was associated with a 51% lower risk of depression (odds ratio: 0.51, 95% CI: 0.40–0.64). Subgroup analysis revealed that this association was particularly pronounced among adults aged 40 and older, as well as non-Hispanic whites and non-Hispanic blacks.

**Conclusion:**

Encouraging outdoor activities may represent an effective public health strategy to reduce depression risk, particularly among middle-aged and older adults and specific ethnic populations. Public health policies should prioritize initiatives that encourage outdoor engagement, and future research is needed to explore the underlying mechanisms and population-specific responses to outdoor activity.

## Introduction

Depression is a leading cause of disability worldwide, affecting an estimated 300 million individuals and contributing significantly to global disease burden and economic challenges (Frühauf et al., 2016). In the United States alone, depression impacts nearly 8.4% of adults annually, highlighting the critical need for effective prevention and treatment strategies (Owens and Bunce, 2022).

Among various interventions, outdoor activities have gained attention due to their potential to promote mental health through unique physiological and psychological mechanisms. These include enhancing physical activity, increasing vitamin D synthesis, improving sleep quality, and reducing stress (Hahn et al., 2011). Unlike pharmacological or purely psychological approaches, outdoor activities offer a holistic and accessible option for many populations, making them particularly worthy of focused research.

Previous studies have provided valuable insights into the benefits of outdoor activities. For instance, Frühauf et al. (2016) demonstrated that outdoor exercise significantly improved mood and psychological well-being, outperforming indoor exercise in boosting vitality and positivity (Buckley et al., 2018). Owens and Bunce (2022) further proposed that natural environments enhance mental health by reducing stress, decreasing rumination, and fostering positive thought patterns (Hahn et al., 2011). However, not all studies have reached consistent conclusions, with some failing to identify significant associations (Frühauf et al., 2016). These discrepancies underscore the influence of cultural, environmental, and socioeconomic factors on the effectiveness of outdoor activities (Wicks et al., 2022). Additionally, individual differences such as age and ethnicity may shape responses to outdoor activities. Despite these variations, limited research has systematically explored how these demographic factors impact the relationship between outdoor activity and depression. Addressing this gap is crucial to developing targeted, equitable public health interventions (Zhang, 2017).

This study aims to evaluate the relationship between time spent outdoors and depression among U.S. adults using NHANES 2011–2018 data. By focusing on differences across age and ethnic groups, this research seeks to provide robust evidence for integrating outdoor activities into public health strategies and tailoring interventions to meet the needs of diverse populations (Wheeler et al., 2020).

## Methods

### Study population and data sources

This cross-sectional study utilized data from the National Health and Nutrition Examination Survey (NHANES), a program conducted by the Centers for Disease Control and Prevention (CDC). NHANES uses a stratified, multistage probability sampling design to collect data representative of the civilian noninstitutionalized U.S. population. Oversampling of certain subgroups, such as older adults and minority populations, ensures robust analysis across demographic categories. Detailed sampling methods are available on the NHANES website.^1^

**Figure 1:**
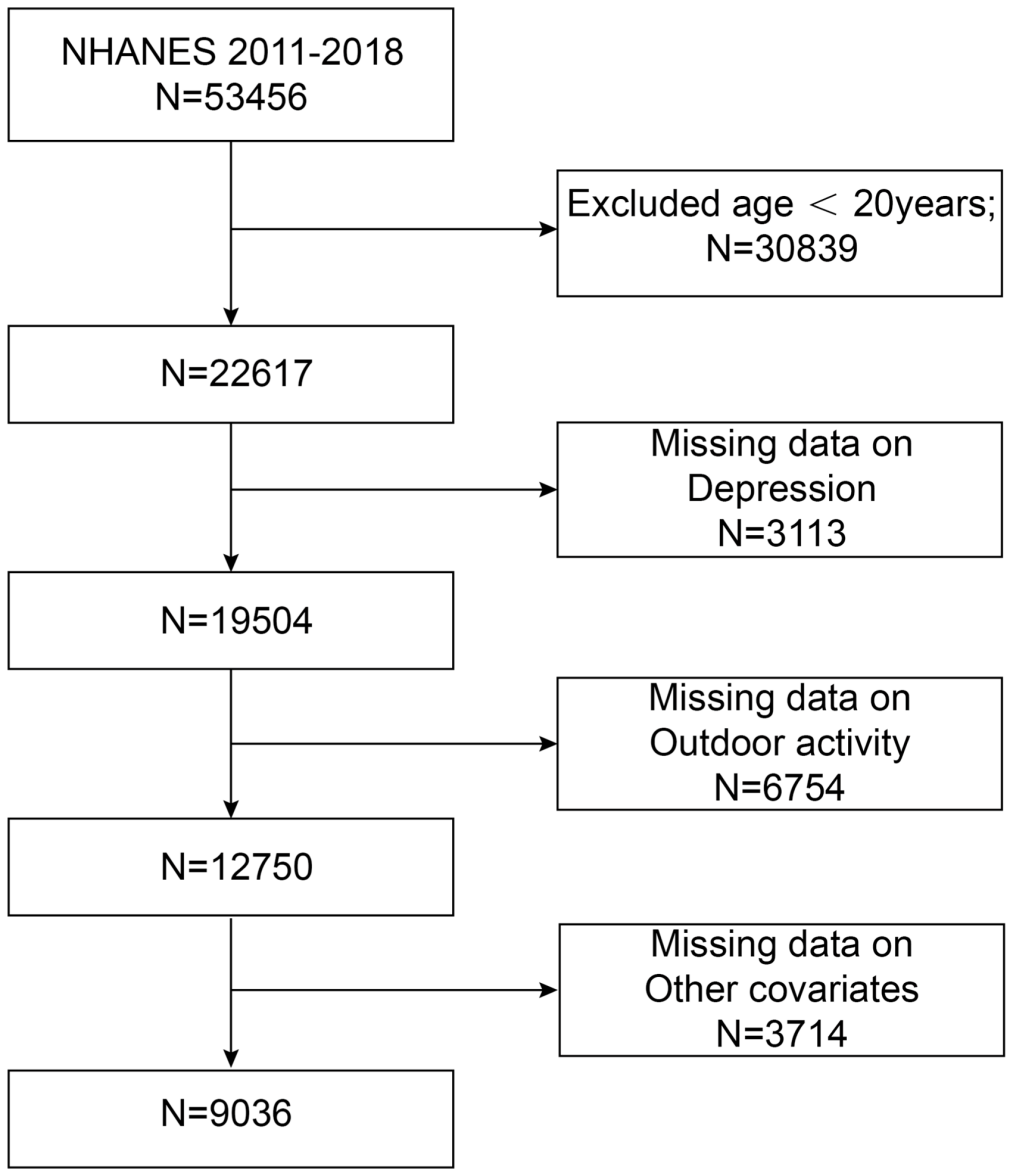
Flowchart of the study population.

We analyzed data from NHANES 2011–2018, which included a total of 53,456 participants. After limiting the dataset to individuals aged ≥20 years, we excluded participants with missing depression scores (*N* = 3,113), outdoor activity data (*N* = 6,754), and other covariates (*N* = 3,714), resulting in a final analytical sample of 9,036 participants. The participant selection process is outlined in Figure 1.

### Variables in research

#### Outdoor activity

Outdoor activity time was measured using the Physical Activity Questionnaire, derived from the Global Physical Activity Questionnaire (GPAQ). Participants reported durations of various outdoor activities, such as walking, jogging, gardening, and other leisure-time exercises. Data were collected using the Computer-Assisted Personal Interviewing (CAPI) system, with implausible responses (e.g., >24 h/day) excluded during data processing to ensure validity.

#### Depression

Depressive symptoms were assessed using the Patient Health Questionnaire (PHQ-9), a validated tool consisting of nine questions on symptom frequency over the past 2 weeks. Responses ranged from 0 (“not at all”) to 3 (“nearly every day”), with total scores (0–27) indicating severity. Interviews were conducted privately using CAPI in English or Spanish to reduce recall and reporting bias.

#### Covariates

Categorical variables: Gender, race/ethnicity (Mexican American, other Hispanic, non-Hispanic black, non-Hispanic white, or other), education level, marital status, high blood pressure status, diabetes status, smoking status (≥100 lifetime cigarettes or not), and daily alcohol consumption. Continuous variables: Body mass index (BMI, kg/m^2^) and daily sedentary time (minutes). Measurement protocols for all variables are publicly available on the NHANES website.

### Statistical Models

#### Analysis approach

All statistical analyses were performed using R (version 3.4.3) and EmpowerStats software (version 4.1). Continuous variables were expressed as mean ± standard deviation (SD), and categorical variables as percentages. Baseline comparisons were conducted using linear regression models for continuous variables and chi-square tests for categorical variables. To investigate the association between outdoor activity time and depression risk, we developed three models with incremental adjustments to address potential confounding: Model 1 (Unadjusted): This model explored the crude relationship between outdoor activity and depression without adjusting for any confounders. Rationale: Establishes the baseline association and highlights the raw effect size. Model 2 (Partially Adjusted): Adjusted for age, gender, and race/ethnicity. Rationale: These primary demographic factors are strongly associated with both outdoor activity participation and mental health outcomes (U.S. Department of Health and Human Services, 2018). Adjusting for them helps isolate the association between outdoor activity and depression. Model 3 (Fully adjusted): included additional adjustments for education level, marital status, hypertension, diabetes, smoking status, alcohol consumption, BMI, and daily sedentary time. Rationale: these variables represent key health and lifestyle factors that may influence depression risk or participation in outdoor activities. For example, sedentary behavior is inversely associated with outdoor activity and is an independent predictor of depression (Pearce et al., 2022; Ströhle, 2009). Adjusting for these factors allows for a more comprehensive evaluation of the independent relationship between outdoor activity and depression.

#### Subgroup analyses

Subgroup analyses were conducted by gender, age, race/ethnicity, education level, and BMI. BMI was included as a stratification variable due to its dual association with physical activity and mental health, reflecting potential interactions between physical and psychological factors (Firth et al., 2020).

#### Ethics and data collection

The NHANES protocol was approved by the NCHS Ethics Review Board. Written informed consent was obtained from participants or their legal guardians. To minimize recall bias, data collection utilized validated tools (e.g., PHQ-9, GPAQ) and private, standardized interviews conducted via CAPI, ensuring consistent and accurate responses.

## Results

### Baseline characteristics

The baseline characteristics of 9,036 participants are summarized in Table 1. Participants with depression scores ≥10 were more likely to have sedentary time ≥ 480 min per week, outdoor activity <60 min per week, and higher BMI (31.48 ± 8.89 vs. 29.18 ± 7.29, *p* < 0.0001). This group also included a greater proportion of females (64.9%), individuals aged ≥40 years (56.0%), and those with lower educational attainment (25.69% with high school or below).

**Table 1:** Baseline characteristics of the study population stratified according to depression scores.

Characteristics^a^	Q1 (<10) *N* = 8,207	Q2 (≥10) *N* = 829	*p* value
Age (year)			<0.0001
≥ 20, <30	2,144 (26.12%)	164 (19.78%)	
≥ 30, <40	2052 (25.00%)	201 (24.25%)	
≥ 40, <50	1996 (24.32%)	202 (24.37%)	
≥ 50	2015 (24.55%)	262 (31.60%)	
Gender (%)			<0.0001
Male	4,137 (50.41%)	291 (35.10%)	
Female	4,070 (49.59%)	538 (64.90%)	
Race (%)			<0.0001
Mexican American	1,124 (13.70%)	82 (9.89%)	
Other Hispanic	710 (8.65%)	97 (11.70%)	
Non-Hispanic White	2,994 (36.48%)	367 (44.27%)	
Non-Hispanic Black	1878 (22.88%)	189 (22.80%)	
Other Races	1,501 (18.29%)	94 (11.34%)	
Education level (%)			<0.0001
High school and below	1,350 (16.45%)	213 (25.69%)	
High school and above	6,857 (83.55%)	616 (74.31%)	
Marital status (%)			<0.0001
Married	1822 (22.20%)	151 (18.21%)	
Bereaved of one’s spouse	5,268 (64.19%)	514 (62.00%)	
Divorced	317 (3.86%)	73 (8.81%)	
Unmarried	800 (9.75%)	91 (10.98%)	
Daily alcohol consumption (%)			0.008
Yes	6,112 (74.47%)	652 (78.65%)	
No	2095 (25.53%)	177 (21.35%)	
Smoking over 100 cigarettes (%)			<0.0001
Yes	3,100 (37.77%)	499 (60.19%)	
No	5,107 (62.23%)	330 (39.81%)	
Diagnosis of diabetes (%)			<0.0001
Yes	568 (6.92%)	112 (13.51%)	
No	7,494 (91.31%)	690 (83.23%)	
Borderline	145 (1.77%)	27 (3.26%)	
Diagnosis of hypertension (%)			<0.0001
Yes	1905 (23.21%)	323 (38.96%)	
No	6,302 (76.79%)	506 (61.04%)	
Outdoor activity (min)			<0.0001
<60	1,278 (15.57%)	227 (27.38%)	
≥ 60,<180	2,524 (30.75%)	231 (27.86%)	
≥ 180,<360	2,202 (26.83%)	207 (24.97%)	
≥ 360	2,203 (26.84%)	164 (19.78%)	
sedentary time (min)			0.288
<480	5,114 (62.31%)	501 (60.43%)	
≥ 480	3,093 (37.69%)	328 (39.57%)	
BMI (Kg/m^2^)	29.18 ± 7.29	31.48 ± 8.89	<0.0001

^aWeighted means ± SD for continuous variables; weighted proportions for categorical variables.^

### Association between outdoor activity and depression

**Figure 2:**
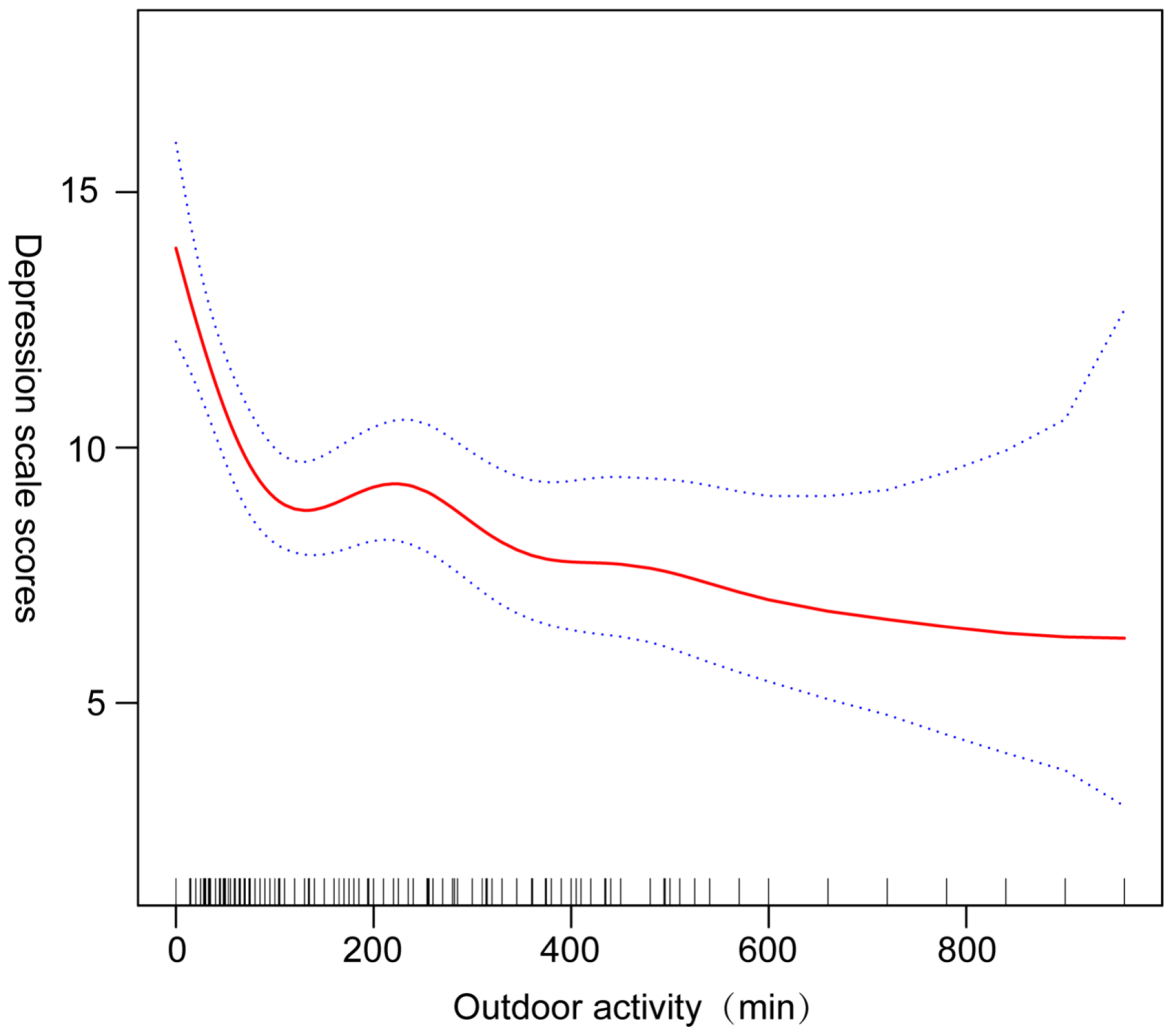
Association of depression with and time spent outdoors (95% CI).

Table 2 presents the relationship between time spent outdoors and depression risk. In the fully adjusted model, participants in the highest quartile of outdoor activity (≥360 min/week) had a 49% lower risk of depression compared to those in the lowest quartile (<60 min/week) (OR = 0.51, 95% CI 0.40–0.64, *p* < 0.0001). A smoothed curve fit (Figure 2) demonstrated a nonlinear negative association between outdoor activity and depression, with diminishing benefits at higher activity levels.

**Table 2:** The relationship between depression and time spent outdoors.

	Crude model	Model 1	Model 2
	OR (95% CI) *p* value	OR (95% CI) *p* value	OR(95% CI) *p* value
Categories (Outdoor activity)			
Quartile 1	Reference	Reference	Reference
Quartile 2	0.52 (0.42, 0.63) <0.0001	0.52 (0.42, 0.63) <0.0001	0.60 (0.49, 0.74) <0.0001
Quartile 3	0.53 (0.43, 0.65) <0.0001	0.56 (0.46, 0.69) <0.0001	0.67 (0.54, 0.83) 0.0003
Quartile 4	0.42 (0.34, 0.52) <0.0001	0.49 (0.39, 0.61) <0.0001	0.51 (0.40, 0.64) <0.0001

Crude model: no covariates were adjusted.

Model 1: age, sex, race were adjusted.

Model 2: Fully adjusted models.

### Subgroup analyses

**Figure 3:**
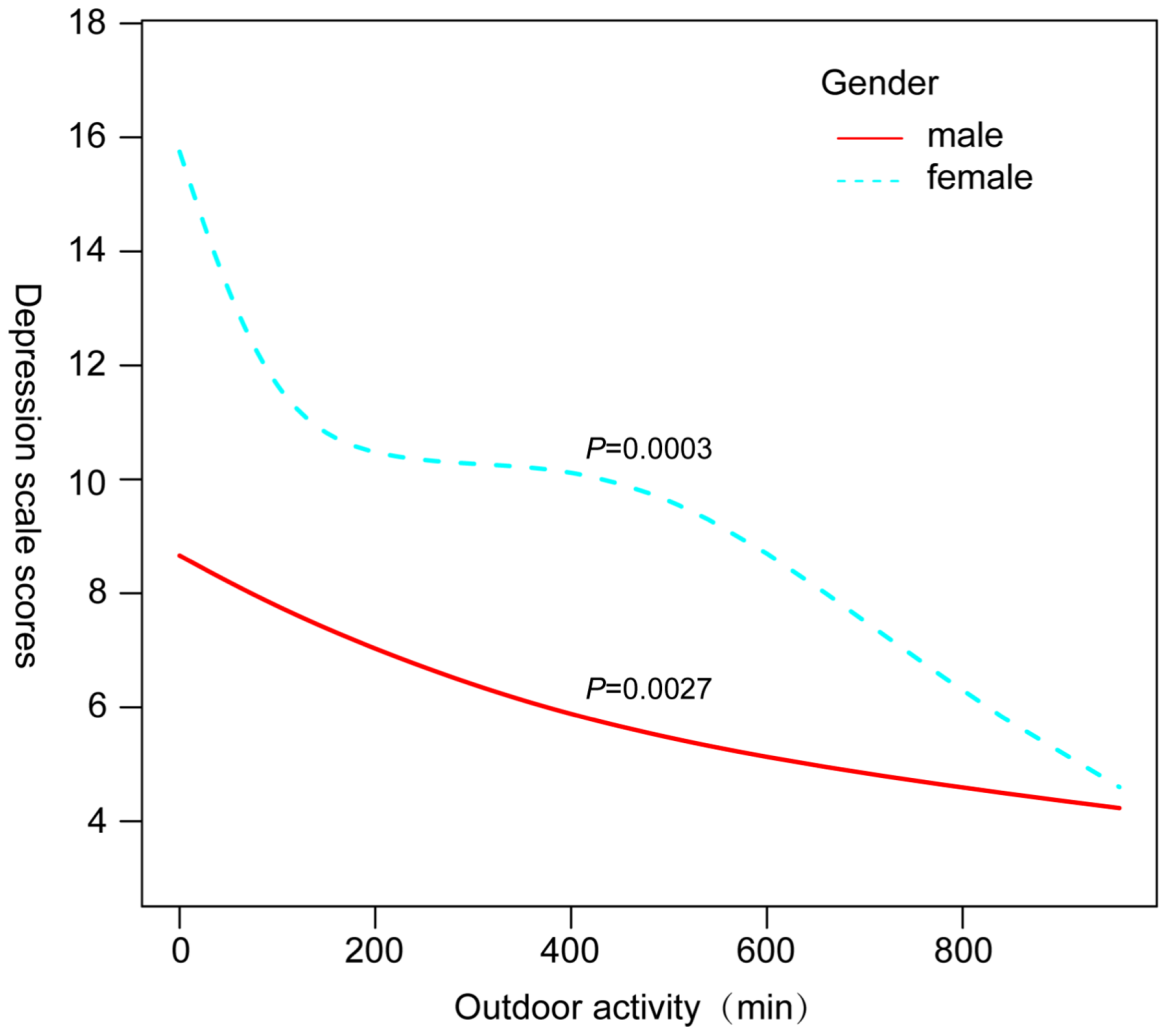
Smoothed curve fitting plots stratified by gender.

**Figure 4:**
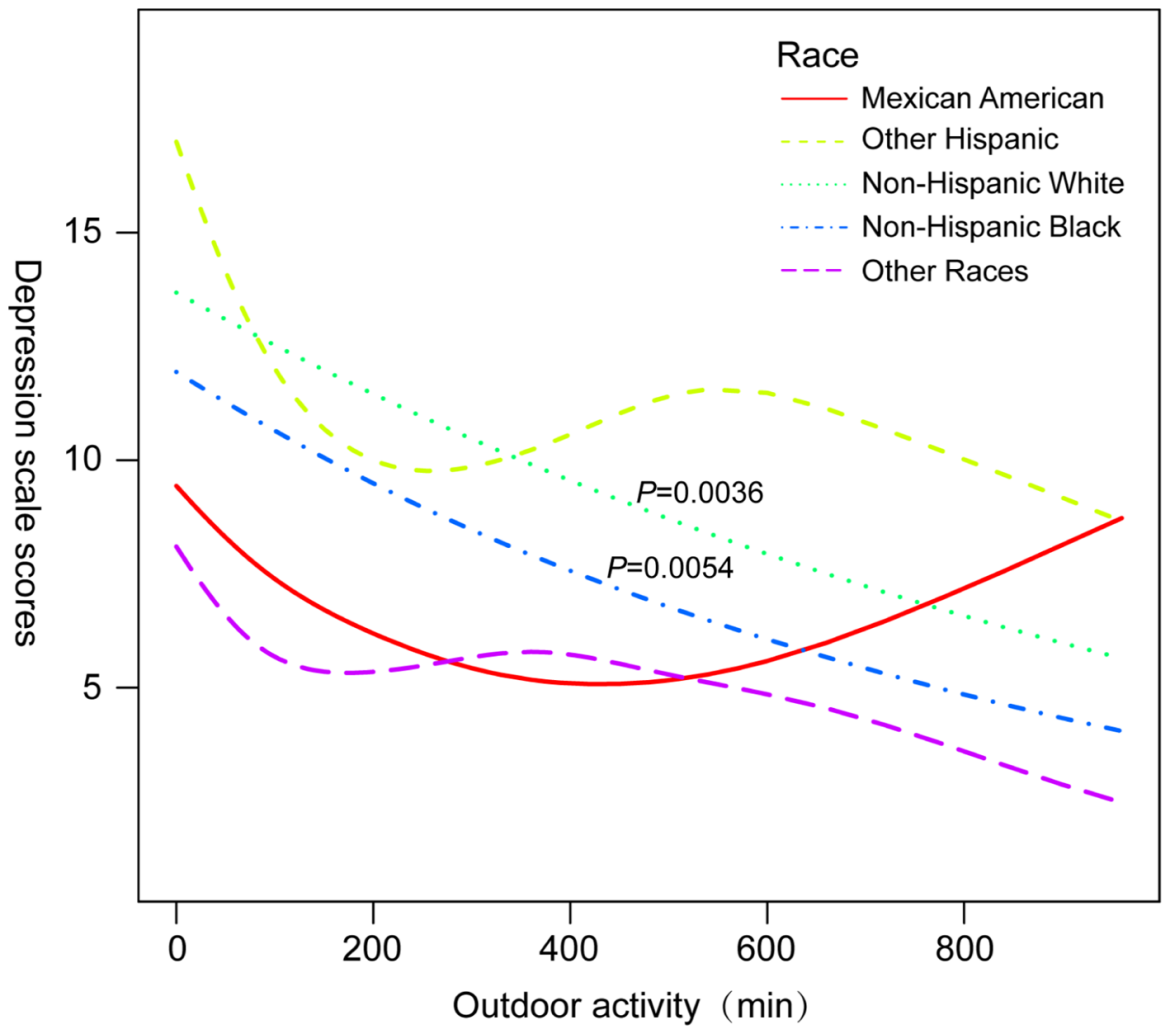
Smoothed curve fitting plots stratified by race.

**Figure 5:**
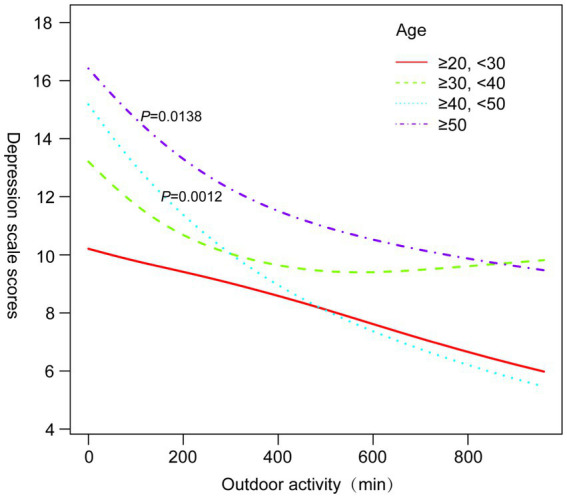
Smoothed curve fitting by age stratification.

**Figure 6:**
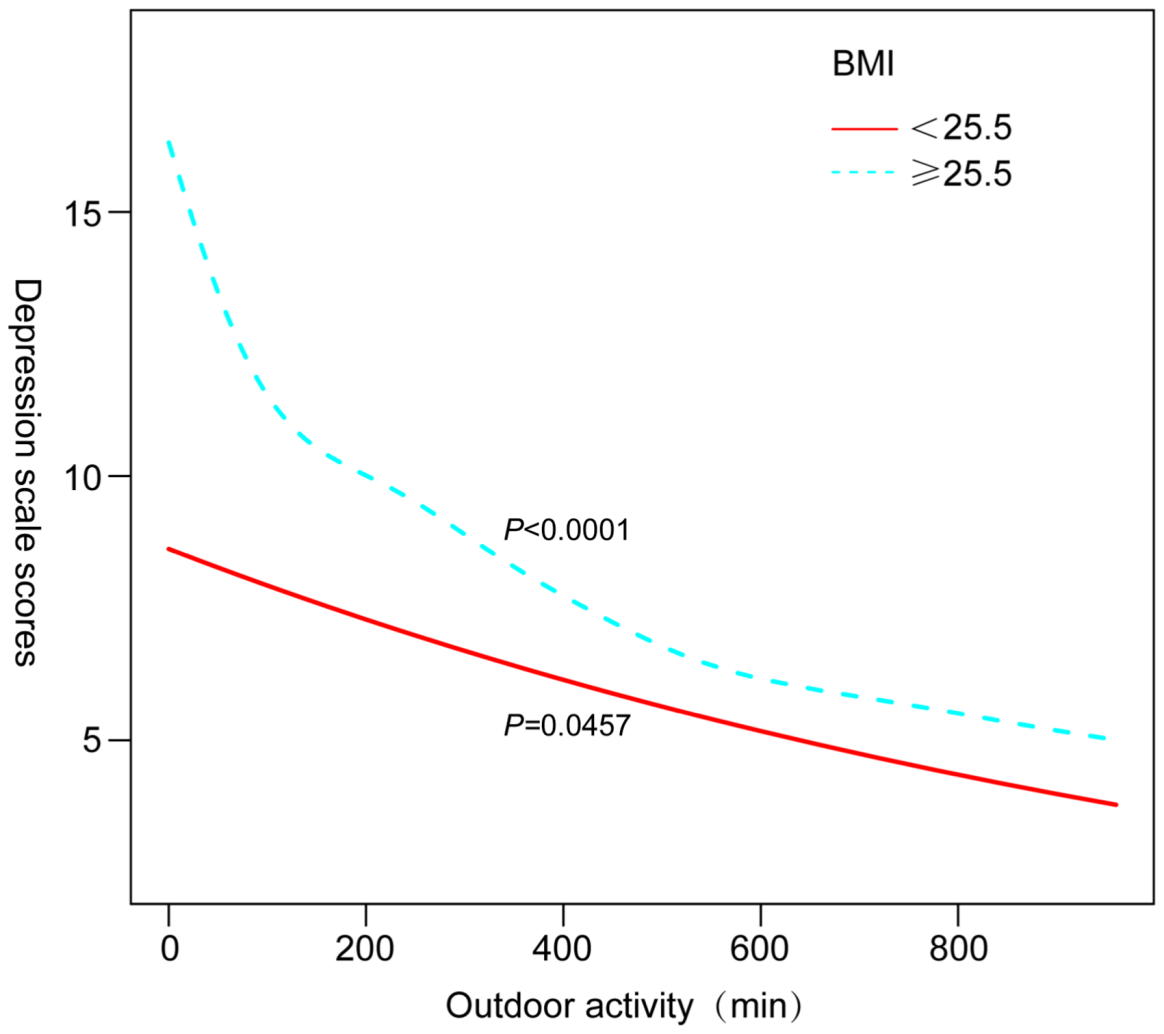
Smoothed curve fit stratified by BMI.

Subgroup analyses (Table 3) revealed that the protective association between outdoor activity and depression was particularly significant among participants aged ≥40 years (OR = 0.67, 95% CI 0.54–0.83, *p* = 0.0003), non-Hispanic whites (OR = 0.42, 95% CI 0.34–0.52, *p* < 0.0001), and those with BMI ≥25.5 kg/m^2^ (OR = 0.60, 95% CI 0.49–0.74, *p* < 0.0001). Figures 3–6 illustrate these subgroup trends with annotations highlighting significant differences and key findings. Increasing outdoor activity time, especially for middle-aged and older adults, non-Hispanic whites, and individuals with higher BMI, could be a key strategy in reducing depression risk. Public health policies should prioritize promoting outdoor activities tailored to high-risk populations.

**Table 3:** Subgroup analysis.

SII		OR (95% CI)	*P* for interaction
Gender			0.4164
Male	*N* = 4,428	1.00 (1.00, 1.00)	0.0027
Female	*N* = 4,608	1.00 (1.00, 1.00)	0.0003
Age(years)			0.5096
≥20, <30	*N* = 2,308	1.00 (1.00, 1.00)	0.1958
≥30, <40	*N* = 2,253	1.00 (1.00, 1.00)	0.0641
≥40, <50	*N* = 2,198	1.00 (1.00, 1.00)	0.0012
≥50	*N* = 2,277	1.00 (1.00, 1.00)	0.0138
Race (%)			0.4242
Mexican American	*N* = 1,206	1.00 (1.00, 1.00)	0.3783
Other Hispanic	*N* = 807	1.00 (1.00, 1.00)	0.1754
Non-Hispanic White	*N* = 3,361	1.00 (1.00, 1.00)	0.0036
Non-Hispanic Black	*N* = 2067	1.00 (1.00, 1.00)	0.0054
Other Races	*N* = 1,595	1.00 (1.00, 1.00)	0.1457
BMI(Kg/m2)			0.3672
<25.5	*N* = 2,992	1.00 (1.00, 1.00)	0.0457
≥25.5	*N* = 5,995	1.00 (1.00, 1.00)	<0.0001

## Discussion

This study explored the relationship between outdoor activity duration and depression among U.S. adults using NHANES 2011–2018 data. Our findings revealed a significant negative association between outdoor activity time and depression risk. Fully adjusted models indicated that for each additional minute spent outdoors, the risk of depression decreased by 0.51-fold (Kerr et al., 2012). Subgroup analyses further demonstrated that this association was particularly strong among individuals aged 40 years and older, non-Hispanic whites, and non-Hispanic blacks.

Recent studies further substantiate the positive association between outdoor activity and mental health. For instance, Kerr et al. (2012) demonstrated that outdoor physical activities significantly alleviated depressive symptoms among middle-aged adults. Similarly, Hartig et al. (2014) highlighted the role of nature-based activities in improving mental well-being, particularly during post-pandemic recovery periods. Moreover, Pretty et al. (2005) emphasized the importance of integrating green exercise into community programs to address mental health challenges. These findings underscore the necessity of tailoring interventions to different population needs and highlight the evolving landscape of mental health research.

Mechanistically, the observed association between outdoor activity and reduced depression risk can be explained through several biological and behavioral pathways. Outdoor activity increases exposure to sunlight, which promotes vitamin D synthesis—a critical factor in mood regulation. Vitamin D deficiency has been strongly associated with depressive symptoms in numerous studies (Holick, 2007). Physical activity, often a component of outdoor engagement, elevates endorphin levels, reduces inflammation, and mitigates oxidative stress, all of which improve mood (Pearce et al., 2022; Ströhle, 2009). Additionally, time spent in natural environments has been shown to reduce cortisol levels and enhance parasympathetic activity, fostering relaxation and stress reduction (Ulrich, 1991). Regular outdoor activities also improve sleep quality, which is essential for maintaining emotional balance and mental health (Baglioni et al., 2011; Passos et al., 2011).

While most studies support the positive effects of outdoor activities, some have found no significant association. For example, a study based on Swedish adults found that, despite physical health benefits, outdoor activities did not significantly reduce depressive symptoms (Mitchell and Popham, 2008). This inconsistency could stem from cultural and environmental differences, as well as disparities in socioeconomic factors influencing both participation and effectiveness of outdoor activities (Firth et al., 2020). Individual variability, including genetic predisposition and lifestyle differences, may also play a role, requiring further research to clarify these interactions (Richardson et al., 2005).

Despite the valuable insights provided by this study, certain limitations must be acknowledged. First, the cross-sectional design precludes inference of causality. Future research should adopt longitudinal or experimental designs to establish causality. Second, self-reported measures of both outdoor activity and depressive symptoms may introduce recall bias and social desirability bias. Third, we did not account for confounders such as seasonal variation, geographic differences, or accessibility to outdoor spaces, which may have influenced the results. Furthermore, individual factors like genetic predispositions and specific health conditions were not controlled for, which may limit the generalizability of findings (Holick, 2007).

To address these limitations, future studies should focus on longitudinal designs that track outdoor activity and mental health outcomes over time. Randomized controlled trials examining specific types and intensities of outdoor activities could also help identify the most effective interventions. Moreover, studies exploring cultural and environmental factors that shape the relationship between outdoor activity and depression will enable the development of tailored public health strategies. Finally, research on population-specific responses to outdoor activity, including those influenced by genetic and socioeconomic factors, will further enhance the practical application of these findings (Richardson et al., 2005).

## Conclusion

This study demonstrates that increased time spent outdoors is significantly associated with a reduced risk of depression, particularly among middle-aged and older adults, non-Hispanic whites, and blacks. These findings emphasize outdoor activity as a practical and accessible intervention to enhance mental health. Incorporating outdoor engagement into urban planning, community initiatives, and clinical practice could provide meaningful benefits, especially for populations at higher risk of depression.

Future research should explore causal relationships through longitudinal studies and randomized trials, while investigating the optimal types, intensities, and durations of outdoor activities to tailor interventions for diverse demographic and cultural contexts.

## Data Availability

Publicly available datasets were analyzed in this study. This data can be found at: https://www.cdc.gov/nchs/nhanes/irba98.htm.

## Footnotes

^1^ https://www.cdc.gov/nchs/nhanes/index.htm
